# Mesenchymal stem cells (MSCs) as skeletal therapeutics–an update

**DOI:** 10.1186/s12929-016-0254-3

**Published:** 2016-04-16

**Authors:** Hamid Saeed, Muhammad Ahsan, Zikria Saleem, Mehwish Iqtedar, Muhammad Islam, Zeeshan Danish, Asif Manzoor Khan

**Affiliations:** Section of Clinical Pharmacy, University College of Pharmacy, University of the Punjab, Allama Iqbal Campus, 54000 Lahore, Pakistan; Department of Bio-technology, Lahore College for Women University, Lahore, Pakistan; Department of Biochemistry and Molecular Biology, University of the Southern Denmark, 5230 Odense, Denmark

**Keywords:** MSCs, Autologous, Osteogenic imperfecta, Infantile Hypo-phosphatasia, Craniofacial, Vertebral disc, Cartilage repair, Bone fracture, Osteoporosis, Osteo-arthritis

## Abstract

Mesenchymal stem cells hold the promise to treat not only several congenital and acquired bone degenerative diseases but also to repair and regenerate morbid bone tissues. Utilizing MSCs, several lines of evidences advocate promising clinical outcomes in skeletal diseases and skeletal tissue repair/regeneration. In this context, both, autologous and allogeneic cell transfer options have been utilized. Studies suggest that MSCs are transplanted either alone by mixing with autogenous plasma/serum or by loading onto repair/induction supportive resorb-able scaffolds. Thus, this review is aimed at highlighting a wide range of pertinent clinical therapeutic options of MSCs in the treatment of skeletal diseases and skeletal tissue regeneration. Additionally, in skeletal disease and regenerative sections, only the early and more recent preclinical evidences are discussed followed by all the pertinent clinical studies. Moreover, germane post transplant therapeutic mechanisms afforded by MSCs have also been conversed. Nonetheless, assertive use of MSCs in the clinic for skeletal disorders and repair is far from a mature therapeutic option, therefore, posed challenges and future directions are also discussed. Importantly, for uniformity at all instances, term MSCs is used throughout the review.

## Background

Bone marrow stroma consists of heterogeneous cell populations that assist the processes of bone homeostasis and hematopoiesis [1, 2]. After hematopoietic stem cells, among the stromal cell populations, bone marrow mesenchymal/stromal stem cells (MSCs)–the non-hematopoietic portion, are the second most extensively studied population [3].

### Mesenchymal stem cells (MSCs)

In 1970, Friedenstien first reported the presence of non-hematopoietic stem cell population in bone marrow stroma by culturing whole bone marrow in a culture dish and by removing non-adherent cells–leaving behind the adherent cells with fibroblast like morphology, capable of forming discrete colonies and exhibit density insensitive growth [4]. Initially, MSCs were studied because of their pivotal role in creating hematopoietic supportive micro-environment, but later came to prominence owing to their role as precursors of skeletal tissue/bone [5–7]. Friedenstein and colleagues were the first to demonstrate the osteogenic potential of cells obtained from the bone marrow stroma with stemness characteristics [8].

### Multi-lineage potential and pertinent tissue sources of MSCs

Studies have demonstrated that these MSCs have the ability to self-renew and are multi-potent in nature, meaning that they can be expanded and form discrete colonies of undifferentiated cells, yet retain the ability to differentiate into different mesenchymal lineages such as, osteoblasts, adipocytes and chondrocytes [9–12]. Further studies revealed that MSCs can differentiate into other lineages i-e., neurons, skeletal muscle [13] and myocardium [14]. However, only small numbers of these cells (MSCs) can be obtained from the bone marrow, accounting for about 0.001−0.01 % of the total bone marrow cell population [10].

### Characterization of MSCs

MSCs are characterized by the identification of surface antigens/markers. In this regard, numbers of surface markers (CD markers) have been identified to distinguish a stem cell population from other cell types in various cellular compartments, including bone marrow. There is a considerable accord that MSCs are negative for hematopoietic surface markers: CD34, CD45, CD14 and positive for: *STRO*-*1*, CD29, CD73, CD90, CD105, CD106, CD166, CD146, and CD44 [15, 16]. Among these, some are used individually or in combination in an attempt to obtain a more homogeneous population. For example, *STRO*-*1* alone or in combination with CD106 (*VCAM*-*1*), CD146 (*MUC18*) [17], CD271 (low affinity nerve growth factor receptor) [18], CD18 (b-2 integrin) [19] and embryonic stem cell marker; SSEA-4 [20] has been employed for enrichment purposes. More recently, several other markers were employed, alone or in combination, to document their in vivo location and nature, such as PDGFR-α, Nestin and α-SMA [2, 21, 22]. Though all these putative markers identified self-renewing multi-potent MSCs like populations, yet, the scientific community still lack considerable agreement on a set of reliable and definitive markers that define their in vivo nature and origin, thus, there is a need to identify a more stringent and definitive set of markers to identify MSCs in vivo.

### Are MSCs isolated from various tissues the same?

Since a very low number of MSCs can be isolated from bone marrow aspirates, attempts have been made to isolate MSCs like cells from other tissues, i-e., peripheral blood [23], adipose tissue [24], umbilical cord blood [25], synovial membranes [26], deciduous teeth [27], liver [28, 29] and amniotic fluid [30]. However, despite sharing some common properties (surface markers), as enlisted by International Society for Cellular Therapy (ISCT) guidelines, these various MSCs populations exhibited differences in their differentiation potential and gene expression profiles, when compared alongside [31]. Since their identification, significant differences among MSCs isolated from various tissues have been reported, such as differences in relative ease of propagation, differentiation spectrum and expression of cell surface markers (*STRO*-*1*, SSEA-4, CD27 and CD34) [32, 33]. Regarding the superiority of the isolate, Yoshimura et al., demonstrated that rat synovial MSCs (S-MSCs) are superior than bone marrow, adipose, periosteum and muscle derived MSCs in terms of colony and cell numbers coupled with low degree of invasiveness, sans any complications at the donor site [34]. Furthermore, scientific community lacks considerable agreement regarding the molecular signature (surface markers) in original stromal vascular fractions and later during culture expansion. In this regard, studies have shown that surface markers expression is heterogeneous in original stromal vascular fractions, such as low expression of CD54, CD31 and CD34, followed by rapid transition to a more homogeneous expression of CD 29, CD73, CD90 and CD105 [35]. However, the detailed description of differences in MSCs populations, isolated from various tissue compartments, is beyond the scope of this review.

Thus, further in this manuscript, we discussed pertinent therapeutic mechanisms of MSCs in skeletal repair and regeneration followed by therapeutic uses of MSCs in skeletal diseases and skeletal tissue repair/regeneration.

### Afforded mechanisms of MSCs in skeletal disease and tissue repair/regeneration

Mesenchymal stromal stem cells (MSCs) have been in the clinical settings for the last 10 years. Thus, plethora of literature reports were aimed at delineating the probable mechanisms through which MSCs afford their clinical attendance.

MSCs produce their tissue reparative role by migration to the site of injury upon receiving specific signals [36]. In this context, several chemotactic factors, receptors and growth factors have been identified utilizing diverse tissue specific regenerative themes involving skeleton, brain, liver and heart [37]. Notable chemotactic factors/receptors reported so far include, SDF1-CXCR4 axis, CX3CL1-CX3CR1 axis and LPA-LPA1 axis [38, 39]. Similarly, noteworthy cytokines include, IL6, TNF-a, and IL1b, while participating growth factors are IGF1, PDGF-BB, TGF-b and HGF [36, 38]. With the release and expression of these signals, the circulating MSCs get entrapped within the tissue vasculature, thus setting a platform for MSCs homing which subsequently proliferate to offer specialized progeny vital for tissue repair and regeneration [37, 40]. Despite considerable understanding, the exact mechanisms of MSC homing to injured tissue are still obscure.

Similar to migration, homing is dependent upon the migratory abilities of MSCs to home at the site of injury after transplantation. Homing is a multistep process-involving cascade of events; nevertheless, the most critical step is the rolling ability of MSCs mediated by expression of receptors on the circulating cells with subsequent engagement of relevant endothelial receptors apt for tethering and cell rolling contacts with endothelium, followed by activation of integrin base adhesiveness vital for adhesion of implanted cell to the extracellular matrix of target tissue/organ [41].

Another trademark of MSCs signifying its clinical value in improved therapeutic outcomes, after transplantation, is the induction of angiogenesis. Number of preclinical and clinical studies has demonstrated the role of MSCs in promoting angiogenesis by virtue of VEGF, HGF, FGF2 and angiogenin [42, 43]. A very few preclinical literature evidences exist that demonstrated the role of MSCs in bone repair via angiogenesis. In this context, Li et al., provided first preclinical evidence of MSCs driven angiogenesis in rabbit avascular femoral head necrosis model. Data demonstrated that intravenous injection of allogenic MSCs resulted in vascular and bone regeneration attributed to the production of BMP, VGEF and OPN [44]. More recently, MSCs have been genetically modified to produce osteogenic and angiogenic growth factors, thus MSCs act as indigenous factories to produce desired factors in a spatial and temporal fashion–promoting bone regeneration [45].

Previously, it was a predominant belief that MSCs exert their therapeutic effect by replacing damaged tissue and promoting tissue regeneration [46]. Recently, several lines of evidence suggested that administered MSCs mediate their tissue protective effect mainly by soluble paracrine factors or trophic factors [47–49]. Presumably, spatial and temporal release of these soluble factors is influenced by injured tissue microenvironment [49, 50]. Several of these factors secreted by MSCs are critical mediators of angiogenesis and anti-apoptosis, for example, VGEF, IGF1, bGFG, HGF, IL6 and CCL2 [51, 52]. Similarly, anti-inflammatory effects of MSCs are ascribed to their immune modulatory properties; presumably by modulating inflammation associated immune cells [53]. Furthermore, a pre-clinical study demonstrated that after MSCs administration, serum circulating levels of pro-inflammatory cytokines, such as IL6 and TNF-a, were reduced with concomitant up-regulation in serum circulating levels of IL10, an anti-inflammatory cytokine [54].

Another important way by which MSCs take part in tissue reparative process is via immunomodulation. MSCs have been proposed to act by inhibiting the process of differentiation of monocytes to dendritic cells thereby preventing the presentation of the antigen to T-cells [55]. Moreover, MSCs directly interrupt the proliferation of T-cells by interfering with their division at the G0/G1 cell cycle phase, opposing the actions of interleukin (IL)-2.

Furthermore, MSCs immune modulatory effects are mediated by acclimatizing natural killer cells to a tissue microenvironment favorable for tissue repair and less vulnerable to autoimmune rejection [56]. As per the published data, MSCs have the ability to escape T cell mediated lysis [57], however, the effects are not limited to T cells, ensuing studies further demonstrated that MSCs can inhibit B cell proliferation, suppress natural killer (NK) activation and modulate cytokine secretion profiles by macrophages and dendritic cells [58, 59]. Furthermore, of note, secreted prostaglandin E2 (PGE-2) is considered a chief mediator in mediating most of the immune modulatory effects by MSCs, such as anti-proliferative effects on T and NK cells and modulating soluble factors released by macrophages and dendritic cells [54, 60].

### Therapeutic applications of MSCs in skeletal diseases

**Fig. 1:**
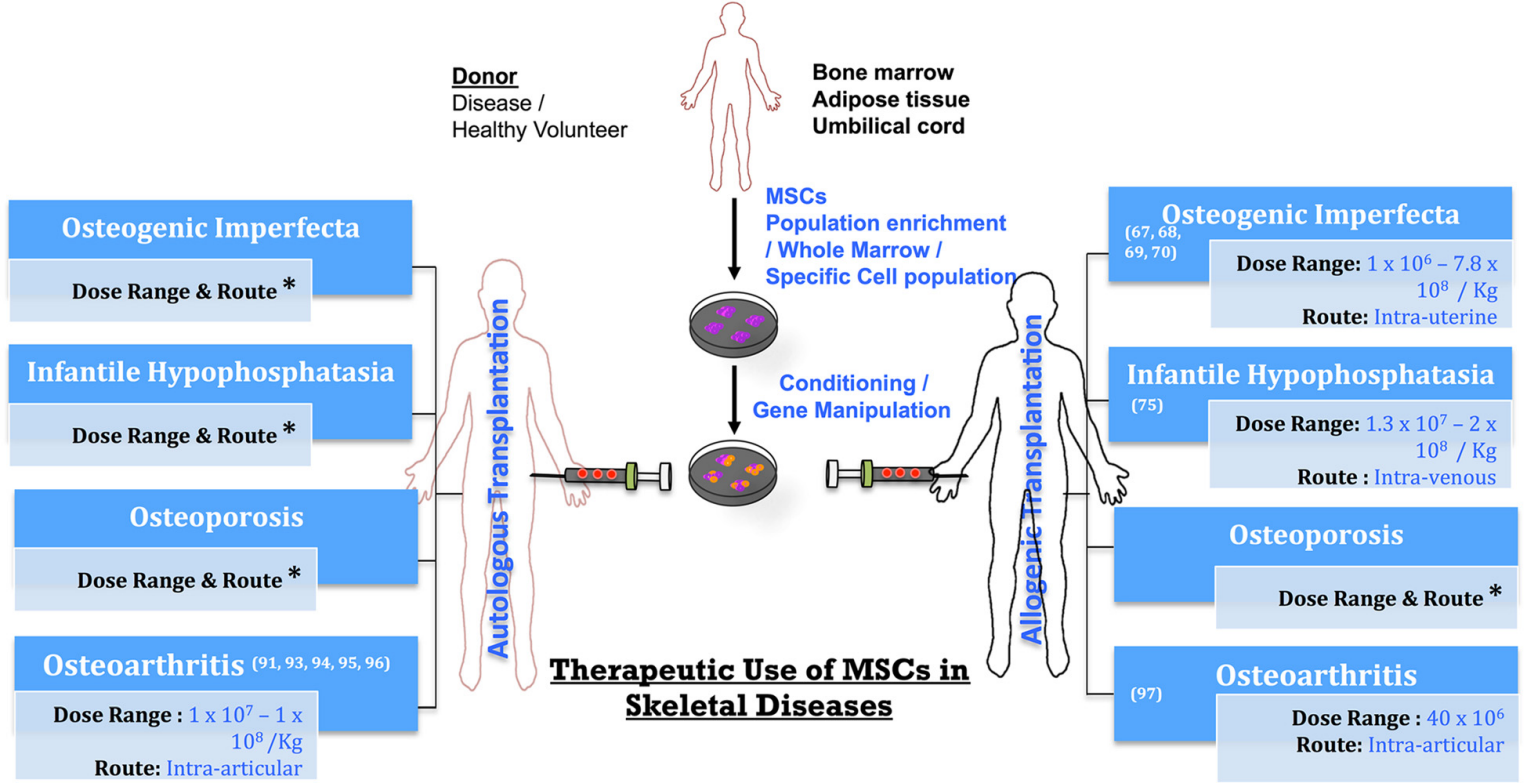
Therapeutics Use of Mesenchymal Stem Cells (MSCs) in Skeletal Diseases. * Indicates that, so far, there is no clinical evidence of autologous cell transplantation after disease gene correction and regarding cell therapy dose and route

A brief overview of skeletal disease related therapeutic options offered by MSCs is shown in Fig. 1, while list of clinical studies employing MSCs in the treatment of skeletal diseases are listed in Table 1. In each type of the disease, under review, only first and more recent preclinical reports were discussed, followed by all the pertinent human studies available to date.

**Table 1:** Clinical Studies Utilizing MSCs in the Treatment of Skeletal Disease

References	Sample size	Cell type	Delivery route	Treatment outcomes
Osteogenesis Imperfecta				
Horwitz et al., 2001 [67]	3	5.5-6.2 × 10^8^ cells per Kg, Allogeneic bone marrow stromal cells (MSCs)	Transplantation	Total body bone mineral content improved from 45 % to 77 % above baseline. Growth velocity improved. The rate of fractures reduced as documented by radiographs.
Horwitz et al., 2002 [68]	6	1-5 × 10^6^ cells per Kg, MSCs transduced with retroviruses	Intravenous infusions, two doses with 8–21 days apart	Patients experienced significant improvement in growth velocity without concomitant increase in bone mineral content
Le Blanc et al., 2005 [69]	Prenatal female fetus	6.5 × 10^6^, HLA mismatched fetal MSCs	Intra-uterine injection	X-ray absorptiometry showed 48 % skeletal mineralization compared to age matched counterpart
Götherström et al., 2014 [70]	Female fetus with type IV OI	30 × 10^6^ cells per kg followed by postnatal dose of 10 × 10^6^, Human fetal MSCs	Intrauterine implantation at 31 weeks of gestation. Thereafter, i.v infusion at 13 month age	Patient was followed for her normal growth trajectory with no alloreactivity from received MSCs
Infantile Hypophosphatasia				
Whyte et al., 2003 [75]	8 mo old girl	2.1 × 10^6^ followed by SCB of 2.92 × 10^7^ mononuclear cells per Kg recipient weight, Haplo-identical marrow stromal cells	Bone marrow transplantation	Striking improvements were seen in skeletal mineralization soon after SCB
Cahill et al., 2007 [72]	8 mo old girl	Four bone fragments (2 mm × 10 mm) + MSCs	Two fragments intraperitonealy and two subcutaneously	Bone mineral contents were increased upto 46 % revealed by x-ray absorptiometry. No change in serum alkaline phosphatase levels was observed
Osteoporosis				
Stenderup et al., 2001 [76]	13	1 × 10^5^ cells per cm^2^, MSCs from Bone marrow aspirate	-	Bone remodeling and turnover occurred at faster rate in osteoporotic patients. However, it was dependent on the continuous availability of osteoprogenitor cells, growth factors and hormones.
Osteoarthritis				
Wakitani et al., 2002 [91]	12	1.3 × 10^7^, Autologous MSCs	Surgical implantation	No significant improvement was seen on clinical evaluation, Histological examination revealed hyaline like cartilage
Centeno et al., 2008 [93]	1	22.4 × 10^6^, MSCs in PBS + PL + dexamethasone	Percutaneous injection	MRI showed improvement in volume of meniscus and cartilage
Pak, 2011 [92]	2	8.3 cm^3^, mixture of Autologous ADSCs, PRP, dexamethasone, hyaluronic acid	Intra-articular injection	At 12 week, significant improvement in pain (more than 90 %) and flexion of knee was experienced by patients. MRI revealed improved cartilage thickness
Davatchi et al., 2011 [94]	4	8-9 × 10^6^, Autologous MSCs	Intra-articular injection	Mild improvement in subjective and objective symptoms was observed.
Orozco et al., 2013 [95]	12	40 × 10^6^, Autologous MSCs	Intra-articular injection	Algofunctional indices strongly indicated clinical efficacy of injected MSCs. T2 mapping demonstrated significantly improved cartilage quality in 11 out of 12 patients.
Jo et al., 2014 [96]	18	1.0 × 10^8^ Adipose tissue derived MSCs	Intra-articular Injection	6 months follow up showed reduction in WOMAC score, MRI findings revealed reduction in the size of cartilage defect
Vega et al., 2015 [97]	15	40 × 10^6^ allogenic MSCs	Intra-articular Injection	Significant improvements in algofunctional indices versus controls and improvements in the quality of cartilage as assessed by T2 measurements

*MSCs* mesenchymal stem cells, *PBS* phosphate buffered saline, *MRI* magnetic resonance imaging, *SCB* stromal cell boost, *HLA* human leukocyte antigen, *PRP* platelet rich plasma *WOMAC* Western Ontario and McMaster Universities Arthritis Index

### Osteogenesis imperfecta

Osteogenesis imperfecta (OI) is a genetic prenatal disorder characterized by osteopenia leading to frequent fractures, bone fragility, bone deformities, and short stature. The underlying cause is the defect in genes (COL1a1, COL1a2) producing type I collagen proteins in osteoblasts [61–63]. Many preclinical studies have indicated the feasibility of transplanting MSCs to treat bony and cartilaginous disorders in animal models of OI [64, 65]. In this regard, Pereira et al. infused MSCs obtained from wild type mice into irradiated transgenic (human mini-*COL1A1*) mice of OI exhibiting fragile bone phenotype. The results of in-situ PCR showed statistically significant increase in bone collagen and mineral content along with the presence of donor derived fibroblast like cells in various non-hematopoietic tissue compartments (2 -12 %) including bone, cartilage and calvaria. Similarly, they also conducted a parallel controlled study by infusing the whole marrow cells [WMC] into female mouse. In this case, despite minimal contribution of fibroblasts (4-6 %), bone collagen was improved significantly [66]. Since bone collagen was improved in both MSCs and WMC group, the positive contribution of non-hematopoietic portion in both the groups in terms of clinical improvements is uncertain, also due to imprecise mixing ratio.

### Clinical evidences

The first clinical evidence of allogeneic stem cell transplantation in type III OI came from the seminal work of Horwitz and colleagues–transplanting un-manipulated bone marrow from HLA identical siblings in affected children [61]. The representative histological samples demonstrated new bone formation after three months of engraftment in bone [20, 61]. Moreover, affected children exhibited increase in growth rate and bone mineral content with significant reduction in bone fracture frequencies, despite poor osteoblast engraftment (<2 %) in bone [61]. Seemingly, the analysis of donor cells engraftment, employed in the study, was under represented due to limited number of biopsies for histological analysis, yet, rather than engraftment, the production of *Col1a1* protein having normal proα polypeptide chain might have contributed towards the reduction in bone fracture and improved growth rate. Besides, Horwitz and co-workers performed further studies employing a similar strategy. In ensuing studies of allogeneic bone marrow transplantation, one clinical study found that the affected children (3 out of 5), after 3 months of treatment, showed an increase of 45−77 % in total body bone mineral content compared to controls [67]. Another study employed six children, undergoing BM transplantation, suggested that MSCs infusion is safe and cells do engraft in bone with subsequent increase in growth velocity and mineralization [68]. Likewise, Le et al. in 2005 performed allogeneic transplantation of MSCs, 6.5 × 10^6^ cells derived from HLA mis-matched male, injected via umbilical vein in fetuses at 32nd week of gestation, having intrauterine fractures associated with severe OI. After preterm delivery at 35th week, in a bone biopsy stained for osteocalcin and osteonectin specific probes, targeting centromeric XY-chromosome, 0.3 % of X (17/6000) and 0.3 % of Y (4/1600), the XY donor cells exhibited engraftment. Importantly, data demonstrated the engraftment of MSCs into bone, even in immuno-competent and HLA incompatible clinical situation [69].

More recently, a different approach was used in treating OI patients, i-e., prenatal allogeneic transplantation of MSCs and postnatal boosting with MSCs from the same donor. Data suggested that transplantation of MSCs during prenatal life was associated with engraftment of MSCs in bone and the beneficial effects started to decrease with passing time–attaining original state. Moreover, postnatal boosting (after 8 years) with MSCs resulted in poor engraftment, though with improved linear growth velocity, mobility and fracture incidences [70]. Thus, in conclusion, data from above mentioned studies corroborate and agreed upon one basic point that MSCs clinical use during prenatal and re-use during postnatal life is safe with no overt toxicities. However, despite minute percentages of MSCs, engrafted after allogenic use in either HLA identical or HLA mismatched immuno-competent clinical states, MSCs therapy is associated with significant reduction in fracture frequencies coupled with improved bone growth and mineral content. Nevertheless, the therapeutic efficacy of MSCs therapy is notably affected during postnatal life and is dependent upon various factors, such as, cell dose, cell type, prior conditioning, prior injury and donor age.

### Infantile hypophosphatasia

A rare inherited metabolic disorder of bones characterized by atypical bone formation and significantly low levels of alkaline phosphatase in serum and bone due to loss of function mutation in tissue non-specific alkaline phosphatase (ALP) gene [71, 72], resulting in impaired mineralization of skeletal tissues, causing osteomalacia or rickets [71]. However, the disease became more severe and debilitating if inheritance is autosomal recessive [73, 74].

### Clinical evidences

Literature searches revealed only two clinical trials on patients with Hypophosphatasia (HPP). In this disease, it is particularly important to investigate therapeutic effects of marrow cell transplantation because defect lies in chondrocytes and osteoblasts [71, 72]. In 2003, Whyte and his co-workers performed first clinical trial of T-cell depleted haplo-identical marrow transplantation in 8 months old girl suffering from infantile hypophosphatasia [75]. Three months post-transplantation, she showed signs of clinical improvements in form of skeletal mineralization and healing of rickets, nevertheless, her disease warrant another booster dose of donor derived marrow cells which resulted in clinical and radiological improvements–less widened growth plates, diminished metaphyseal irregularity and improved bone mineralization. Also, the beneficial effects were attributed to the contribution of donor mesenchymal stem cells towards functional osteoblasts and chondrocytes that presumably ameliorated her disease [75]. However, skeletal biopsies were not taken to confirm donor cell engraftment, probably due to nature and severity of the disease. Similarly, in 2007, Cahill et al. broadened our understanding regarding combined therapeutic approach, i-e., donor bone marrow transplantation followed by donor bone fragment insertions in infantile hypophosphatasia (IHPP). They enrolled a nine months old girl with similar IHPP disease pattern as reported previously [72]. Out of six bone fragments harvested from donor, four bone fragments were inserted in patient, 2 intra-peritoneally (ip), 2 subcutaneously, while 2 were used to culture tissue nonspecific alkaline phosphatase (TNSALP) replete osteoblasts for subsequent transplantation. Data demonstrated improved skeletal mineralization with donor cell engraftment detectable even after twenty months of post transplantation [72]. However, they did not observe any systemic improvements in TNSALP and PPi (inorganic pyrophosphate) despite improvements in mineralization, proposing that deliverance of TNSALP and PPi by donor cells at active skeletal sites is more crucial rather than over all corrections in systemic levels. Thus in conclusion, MSCs do engraft and offer specialized cells boosting skeletal regeneration capacity, yet might not be expanded enough to correct the systemic defects, nonetheless, could sufficiently replace deficient proteins required to correct the tissue (skeletal) specific anomaly.

### Osteoporosis

Osteoporosis, a generalized age-related bone disorder, is characterized by reduced bone mass, due to imbalance between bone formation by osteoblasts and bone resorption by osteoclasts, leading to bone fragility and increased risk of fractures [76, 77]. Numerous literature evidences suggest a plausible link between osteoporosis and defects in MSCs proliferation, differentiation into osteoblast and enhanced apoptosis [78–80]. However, there is a dearth of literature evidences regarding the use of MSCs in the treatment of osteoporosis in humans, thus we discussed a recent preclinical study and pertinent clinical studies. Number of studies has reported the use of MSCs in animal models of osteoporosis [79, 81]. More lately, Tan et al. suggested a possible mechanism of age related osteoporosis by comparing MSCs from adult and young rats for their osteogenic potential and reactive oxygen species production after cyclic stress of mechanical loading [82].

### Partial clinical evidence

Stenderup and his co-workers provided the first clinical evidence [76], conducted on twelve females and one male osteoporotic patients, aged 58−83 and 70 years, respectively, regarding the effect of age related osteoporosis on MSC population. MSCs population from osteoporotic and normal subjects was enriched using *STRO*-*1* to compare MSCs for their proliferative and osteogenic potential. Data suggested that the number and proliferative capacity of MSCs remain unchanged between osteoporotic and normal subjects, thus other factors might be responsible for impaired osteoblast functions in osteoporotic patients [77]. Yet, MSCs isolated from osteoporotic patients exhibited marked reduction in their responses, such as recruitment to fracture sites and towards bone anabolic signals–BMP2 and BMP7 [83]. Similarly, MSCs isolated from osteoporotic patients demonstrated reduced responses to mitogenic signals (IGF-1) along with the production of extracellular matrix sans type 1 collagen, promoting adipogenic differentiation of MSCs–fat overload at the expense of bone [84]. Despite, known multi-faceted pathophysiology of osteoporosis ranging from one’s disease state, genetics, age and life style patterns, such as stress, diet and physical activity–the effects of all these factors on patient’s derived MSCs and its impact on cell therapy is still obscure. Seemingly, in osteoporosis, differentiation potential of MSCs remain unchanged, yet their capacity to generate and respond to signals vital for bone formation and induction, respectively, is reduced compared to controls.

### Osteoarthritis

Osteoarthritis [OA], common form of arthritis, is a degenerative disorder of articular cartilage, involving subchondral sclerosis and local synovial inflammation [85, 86]. OA affects mainly elderly population above 65 years of age demonstrating radiographic signs of diseased cartilage [87, 88]. So far, there is no specific treatment available to prevent or reverse the degenerative process. However, current treatment goal is to reduce pain and associated stiffness, which remains undertreated [89]. In this theme, we will focus more on reviewing clinical evidences rather than pre-clinical reports due to sufficient number of available reports–osteoarthritis and cartilage repair.

### Clinical evidences

According to a published report, MSCs obtained from OA patients exhibited marked reduction in proliferative, chondrogenic and adipogenic potential [90]. To date, clinical trials on humans employing MSCs for the treatment of OA are in initial stages, nevertheless, an extensive research is being conducted to investigate their clinical benefits in OA. In 2002, Wakitani et al., in Japan, conducted first clinical trial regarding the use of MSC in OA [91]. A total of 24 patients were recruited with osteoarthritic knees with prior high tibial osteotomy. Out of 24, 12 patients served as a cell free control while other 12 patients received MSCs suspended in collagen gel into the femoral condyle articular defect, later covered by autologous periosteum. Of clinical importance, the arthroscopic examination revealed better outcomes in treated subjects compared to controls. However, the signs of improvements were not clinically significant. Nonetheless, in 2004, the same research group performed further studies on treated patients and later discovered MSCs embedded in collagen gel in the patellae of two transplanted patients. Clinical benefits of the transplanted MSCs were examined six months post-transplantation, whereupon satisfactory outcomes were obtained as per the improvements in pain and mobility–corroborated by arthroscopy findings of improved cartilage repair by transplanted MSCs.

Pak (2011) reported the efficacy of autologous AD-MSCs (adipose derived MSCs) obtained after processing patient lipo-aspirates to transplant into lateral and medial knee joint of OA patients, after mixing with hyaluronic acid, dexamethasone, calcium chloride and platelet rich plasma. Magnetic resonance imaging (MRI), 12 weeks after the transplantation, revealed significant improvement in the meniscus cartilage thickness located on the knee joints with more than 90 % improvement in pain [92]. Centeno et al. demonstrated a clinical case of 46 years old OA patient involving knee, complaining pain and walking disability. Autologous MSCs were implanted percutaneously which resulted in significant meniscus cartilage regeneration, as evident by MRI, better joint mobility and pain settlement [93]. However, in all of the above studies, immune modulatory role was neither discussed nor considered to examine the role of transplanted donor cell in modulating pro-inflammatory cytokines (Il1β, TNF-α etc.) in deterring cartilage damage.

Likewise, Davatchi and co-workers [94] performed a clinical trial on four patients to assess damage reversal effects of transplanted MSCs in knee joints of OA patients. After in-vitro expansion, MSCs were injected into the affected knees of each patient resulting in improved disease symptoms in 75 % of the patients, i-e., ability to climb stairs on visual analog scale. Furthermore, twelve patients with chronic knee pain, refractory to conservative treatment, were treated with autologous expanded MSCs by intra-articular injection (40 × 10^6^ cells). Data demonstrated significant improvements in cartilage quality by T2 relaxation measurements as evidenced by marked reduction in poor cartilage area [95].

More lately, Jo et al. evaluated the efficacy of transplanted AD-MSCs into the knee joints [intra-articular] of 18 OA patients employing three escalating doses - low (1 × 10^7^), mild-dose (5 × 10^7^) and high dose (1 × 10^8^) [96]. All the treatment outcomes were significantly improved in high dose group, such as, Western Ontario and McMaster Universities Osteoarthritis index [WOMAC] score, visual analog scale for pain, radiological and histological outcomes, further corroborated by arthroscopy findings with significant reduction in the size of cartilage defect in medial femoral and medial tibial condyles [96]. A more recent study by Vega et al.- a randomized clinical trial to evaluate the safety and feasibility of using allogeneic MSCs to treat osteoarthritis, conducted on 30 patients, unresponsive to conventional treatments, by dividing into 2 groups-test and control. The test group received allogeneic bone marrow MSCs (40 × 10^6^ cells) by intra-articular injection, while the control group received intra-articular injection of hyaluronic acid at a single dose of 60 mg. Data demonstrated a significant improvements in cartilage quality in MSC treated patients as quantified by T2 relaxation measurements [97]. Yet, none of the studies, mentioned above, examined the donor cell engraftment to debilitating cartilaginous tissue or attempted to study the expression of various cytokines after MSCs transplantation. Therefore, it is not clear whether the beneficial clinical effects are due to donor cell differentiation, paracrine factors or by modulating host derived pro-inflammatory cytokines. However, data do suggest that allogenic MSCs transplantation is a workable option in patients with osteoarthritis.

Regarding cartilage repair, irrespective of OA, Brittberg and co-workers first demonstrated autologous chondrocyte transplantation in cartilage repair, in brief; chondrocytes were obtained enzymatically from healthy biopsy of articular cartilage, expanded in culture followed by periosteal graft placement over the defect and subsequent injection under the periosteal flap. Data demonstrated significant repair of the tissue along with pain reduction and restoration of joint function [98]. With this promising clinical finding, several other researchers follow suit and performed autologous chondrocyte transplantation in cartilage repair with promising outcomes – improved knee condition and function resulting in better quality of life [99–101]. However, this approach has its demerits and limitation, such as limited expansion of cultured chondrocytes. Thus, later attempts were made using bone marrow mesenchymal stem cells (MSCs) to repair articular cartilage defects. In this context, employing autologous MSCs embedded in collagen gel, a successful attempt was made to treat full thickness cartilage defect in femoral condyle of an athlete. Seven months post implantation, arthroscopy revealed smooth tissue formation at the site of injury followed by histological confirmation of the presence of hyaline like cartilage tissue and improvement of clinical symptoms [102]. In ensuing studies, one-step bone marrow derived cell transplantation approach was used in osteochondral lesions [103]. Similarly, other research groups engaged mesenchymal stem cells in reparative therapies. In one study MSCs were seeded onto collagen plate and transplanted under the grafted periosteoum. Six months post transplantation, significant improvement in clinical symptoms was observed which was further substantiated by histology and imaging [104]. Another study by was conducted by Lee and co-workers employing a minimal invasive technique, arthroscopic micro-fracture, to repair cartilage in human knee by injecting MSCs with hyaluronic acid. A total of seventy patients were enrolled and segregated into two arms; proposed (*n* = 35), minimally invasive technique and open technique (*n* = 35). After final follow-up (24.5 months), significant and comparable improvements, evident by IKDC score (International Knee Documentation Committee), Lysholm knee score, SF-36 physical component score and visual analogue score, were observed in both the groups [105]. More lately, Yamasaki and co-workers treated articular cartilage defect of osteoarthritic knee using autologous MSCs suggesting that postoperative clinical assessment scores were better than pre-operative scores, however, the histological examination revealed that the repaired cartilage did not resemble completely with hyaline cartilage [106]. In all the studies discussed above, despite histological analysis via biopsy samples, donor cell engraftment was not examined; nonetheless, it is pertinent to mention that the use of growth factors, morphogens and scaffolds enhancing cartilage differentiation and engraftment is worth investigating. Moreover, use of biodegradable scaffold, platelet rich fibrin, seems promising. Seemingly, the cartilage repair and within group variation among patient therapy outcomes could be attributed to differences in the condition of cartilage defects at the time of treatment, physical numerals of MSCs actually transplanted, age of the patient and in built inter-individual variations. Additionally, it would be interesting to perform a side by side comparison of different protocols and their effects on donor cell survival, differentiation, engraftment and release of trophic factors to identify the most suitable scaffold apt for clinical purpose.

### Therapeutic applications of MSCs in skeletal tissue repair and regeneration

**Fig. 2:**
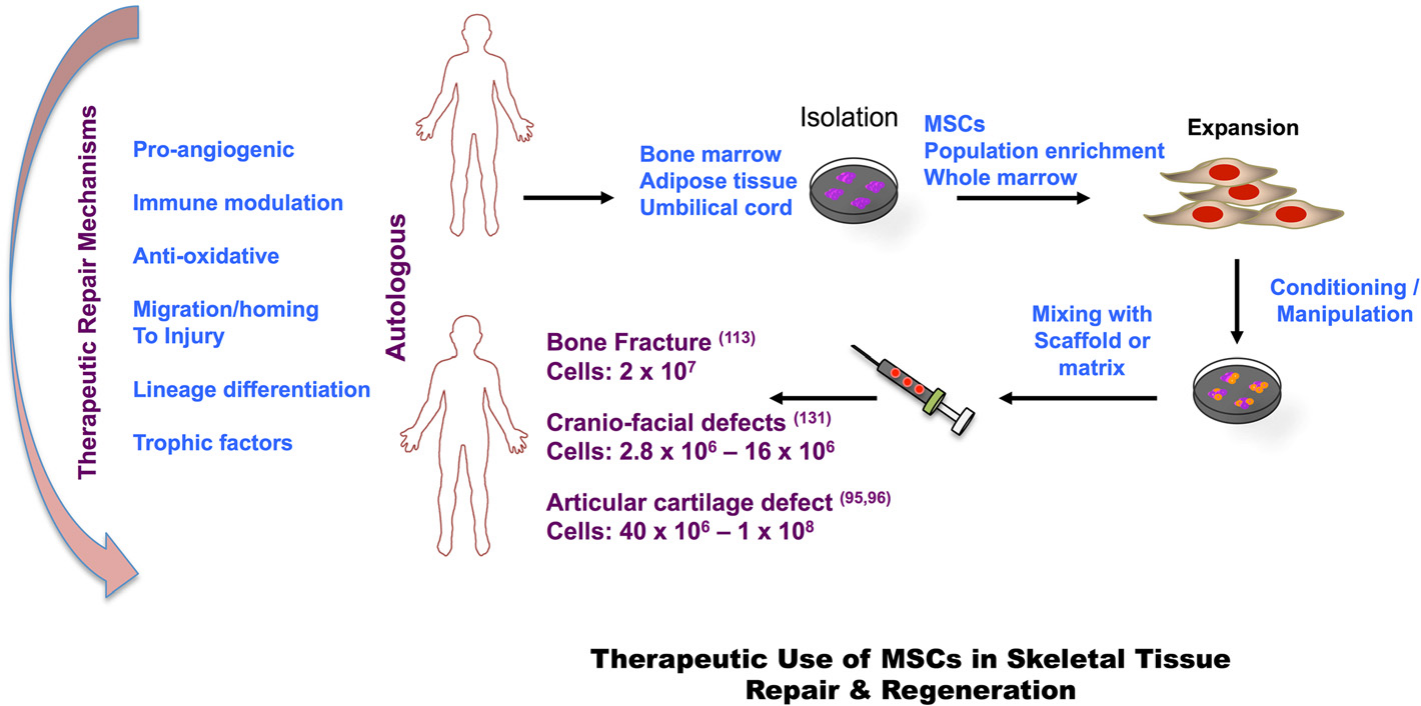
Therapeutics Use of Mesenchymal Stem Cells (MSCs) in Skeletal Tissue Repair and Regeneration

List of clinical studies, reported so far, regarding clinical benefits of MSCs in skeletal tissue repair and regeneration is shown in Table 2. Similarly, macrograph, briefly describing the process, is depicted in Fig. 2. As discussed in disease section, regenerative conditions were reviewed beginning with first and more recent preclinical evidences followed by pertinent clinical studies. Moreover, cartilage regeneration portion has been discussed in Osteoarthritis portion.

**Table 2:** Clinical studies utilizing MSCs for skeletal tissue repair & regeneration

Type of Repair	Studied By	Patients	Cell type	Delivery route	Outcomes
Thoraco-lumbar spine fracture	Faundez et al., 2006 [131]	4	Autologous bone marrow aspirate seeded on MCM coated with hydroxyapatite	Surgical implantation	Both visual and histological analysis confirmed replacement of resorbable matrix with new bone
Mandibular defects	Ueda et al., 2008 [116]	14	3.5 ml of mixture of PRP, MSCs (1.0 × 10^7^cells/ml), 500 μl of thrombin/calcium chloride mixture	Injectable bone grafts via syringes	Successful induction of new bone occurred and osseointegration within short period of time that can reduce the burden of patients
Posterior spinal defects	Gan et al., 2008 [127]	41	MSC suspension	-	Evaluation of patients after 34.5 months showed results of satisfactory spinal in 95.1 % cases
Degenerative disc defects	Orozco et al., 2011 [133]	10	Autologous MSCs	Intra-articular injection	In addition to safety and clinical efficacy, MSCs therapy rapidly settled low back pain and disability with intact normal biomechanics
Anterior maxillary cleft defects	Behnia et al., 2012 [124]	3	MSCs mounted on biphasic scaffolds with PDGF	Surgical implantation	Computed tomography scans showed 51.3 % fill of the bone defect calculated 3 months post-operation
Knee cartilage defects	Lee et al., 2012 [105]	70	Combination of Arthroscopic microfracture, MSCs and hyaluronic acid	Intra-articular injections	Final follow up of 24.5 months revealed considerable improvements in Lysholm knee scale, mean IKDC and visual analog pain scores. Advantages included minimal invasiveness and validated safety
Intertrochanteric Hip fractures	Torres et al., 2014 [115]	15	Autologous bone marrow stem cells concentrate	Surgical implantation	Weight bearing ability of hip and femur improved after 90 days

*IKDC* international knee documentation committee, *MSCs* mesenchymal stem cells, *PDGF* platelet derived growth factor, *PRP* platelet rich plasma, *MCM* mineralized collagen matrix

### Bone fractures

Mesenchymal/marrow stromal stem cells (MSCs) are considered, among others, the attractive therapeutic choices to repair long bone fractures [107]. Plethora of preclinical evidences has demonstrated the reparative effects of autologous or allogeneic MSCs therapy in bone fractures [108–110]. Khadiyala and co-workers reported the first pre-clinical evidence of healing critical size defects employing MSCs. They implanted ex-vivo expanded syngenic MSCs, loaded on to hydroxyapatite/tricalcium phosphate cylinders, into the femora of adult rats that resulted in complete healing of 8 mm segmental defects after 8 weeks [108]. More lately, in rat model of femur fracture, role of MSCs in healing bone fractures was attributed to Sox11 dependent regulation of MSCs differentiation and migration [111].

### Clinical evidences

In clinical settings, Quarto and co-workers contributed first report regarding the use of MSCs in the treatment of large bone defects [112]. They employed a cell based tissue-engineering approach, i-e., osteo-progenitor cell isolation, ex–vivo expansion and subsequent loading on hydroxyapatite scaffolds to treat three patients with large bone defects. All patients demonstrated considerable improvement in limb function supported by clinical evidences obtained via radiographs and computed tomographic scans, revealing callus formation along the implants and appropriate integration at the interfaces with the host bones.

Later studies further demonstrated the promising results of culture expanded autologous osteoprogenitors loaded onto porous HA ceramic scaffolds in the repair of critical size long bone defects [113, 114]. More lately, Torres et al., [115] utilized centrifugation technique to concentrate bone marrow aspirates having 200−2000 mesenchymal stem cells/mL-mixed with hydroxyapatite matrix for implantation into the fracture site. Significant improvement in Harris Hip Scores [HHS] and Visual Analogue Pain Scale (VAS) was observed in the bone marrow concentrate group compared to controls. Above mentioned studies clearly demonstrated that HA scaffold is preferred over other scaffolds, yet the number of progenitor cells and exogenous factors, if any, lack considerable agreement. These findings clearly demonstrate that the transplanted MSCs can contribute towards regeneration of critical size bone defects, however, none of the study demonstrated the contributory percentage of donor and host derived MSCs and process modulation by autocrine and paracrine factors secreted by MSCs.

### Cranio-facial defects

Several craniofacial structures such as the mandibular condyle, calvarial bone and cranial suture have been engineered from mesenchymal stem cells, growth factor, and/or gene therapy approaches [116–118]. In 1998, Krebsbach and his co-workers reported first pre-clinical evidence of bone tissue reparative therapy using mice osseous defect model–transplanting allogeneic MSCs loaded onto gelatin sponges at the site of surgical defects. Data suggested significant repair of surgical defect two weeks after the transplant, attributed to osteogenesis by transplanted MSCs [119]. Recently, Jiang et al., in 2012, employed a different approach in transplanting MSCs to rabbit calvarial defect model, with and without osteogenic induction, by using platelet rich plasma as a scaffold [120]. More lately, another study employing rat calvarial defect model, MSCs (allogeneic) were loaded onto chitosan/alginate/hydroxyapatite scaffold (CAH), with and without BMP-2 impregnation. Data demonstrated significant osteogenic differentiation of MSCs in CAH/BMP2 group corroborated by in vivo findings [121].

### Clinical evidences

In clinical settings, in 2004, Warnke and his co-workers employed bone muscle-flap prefabrication technique to reconstruct and replace subtotal mandible defect. Three-dimensional computed tomography and computer aided design techniques were used to create titanium mesh cage, mimicking subtotal mandibular defect, filled with bone fragments, bone morphogenetic protein 7 and 20 mL of autologous bone marrow aspirate having mesenchymal stem cells. The titanium mesh scaffold was then implanted into latissimus dorsi muscle for 7 weeks–results demonstrated significant re-modeling and mineralization (new bone formation), evident by skeletal scintigraphy and CT scans [122]. Thereafter, more studies were done with promising outcomes using MSCs [123]. In one study autologous MSCs, 5 × 10^5^, mixed with platelet-derived growth factor and mounted on biphasic scaffold–3 mm cubes of HA/TCP, were implanted at the site of alveolar defect, 3 months post-operation, the defect fill was measured around 51.3 % in alveolar pre-maxillary clefts [124]. Furthermore, using slightly different approach of mixed bone marrow cell population (TRCs, tissue repair cells), enriched for CD90 and CD14, Kaigler and co-workers compared guided bone regeneration (GBR) with TRC therapy for the regeneration of craniofacial defect–osseous defect of jaw. Ensuing therapy results demonstrated that osseous defect after tooth extraction exhibited significant regeneration of alveolar bone in TRC group as evident by clinical, histological, tomographic and radiological measures [125]. More recently, Sandor and co-workers reported 13 diverse cases of cranio-maxillofacial hard tissue defects, treated with autologous adipose derived stem cells seeded onto resorbable scaffolds and in some cases impregnated with BMP-2, which resulted in in-situ ossification attributed to adipose derived stem cells [126]. Unlike long bone fractures, in cranio-facial defects, different scaffolds were employed along with different exogenous factors (BMPs, platelet rich plasma and platelet derived growth factor) to promote skeletal tissue regeneration, probably due to the type, shape and size of defects, with the aim to attain specific conformation of a defective portion requiring regeneration. All these studies demonstrate improvement in osseous defects and bone tissue regeneration, yet sans any scientific evidence of the contribution of donor dervied MSCs in bone tissue regeneration or the role of MSCs derived secretory factors in improving bone tissue defects.

### Vertebral disk regeneration

Number of pre-clincal and clinical reports has demonstrated the use of MSCs in the treatment of disc degeneration [127–129]. In this perspective, in a canine disc injury model, Ganey and co-workers demonstrated promising use of ADRCs in disc regeneration, as evident by histology and biochemical analysis, the disc levels of a group, receiving ADRCs with hyaluronic acid, resemble the healthy controls proven by matrix translucency, compartmentalization of the annulus and cell density in nucleus pulposus [130]. Similarly, by transplanting adipose tissue derived stromal stem cells (ADSCs) in rat inter-vertebral degeneration (IVD) model, Jeong et al. demonstrated significant restoration of annulus structure and MRI signal intensity in ADSCs treatment group compared to saline treated group, further corroborated by positive antibody staining for collagen type II and aggrecan of human origin [128].

### Clinical evidences

In clinical practice, Faundez et al., in 2006, first reported the use of autologous bone marrow aspirates impregnated with collagen hydroxyapatite scaffold to produce a bone fusion mass for the treatment of thoracolumbar fracture. After transplantation, significant fusion of bones was confirmed intra-operatively and by mechanical adequacy of fusion mass, which was further substantiated by histological examination of newly formed bone with signs of membranous and enchondoral foci [131]. Furthermore, the first therapeutic intervertebral disc degeneration therapy in humans, employing mesenchymal stromal stem cells, was performed in 2010 by Yoshikawa and his co-workers [132] by enrolling two female patients with lumbar spinal canal stenosis confirmed by myelo-graphy and MRI. Autologous MSCs were expanded ex vivo in autogenous serum and were transplanted percutaneously into the degenerative inter-vertebral discs. Two years post-transplantation, significant improvements were observed in MRI signal intensity, vacuum phenomenon and degenerative symptoms employing radiography and computer tomography [132]. Another pilot study enrolled ten patients with back pain and lumbar disc degeneration, yet with intact annulus fibrosis. Patients were treated with autologous expanded MSCs injected into pulposus area and were evaluated after one year. Results demonstrated rapid improvement of pain and disability within 3 months and water level was elevated at 12 month, though disc height was not improved [133]. Seemingly, culture expanded fraction contains more number of MSCs than freshly isolated bone marrow aspirate, but were less agile. However, the concept has to be confirmed in a side-by-side comparison. Moreover, it’s not clear which type of donor cell contributed towards the improvements, so that a specific cell type can be enriched and expanded ex vivo to achieve more precise therapy outcomes.

### Repair of tendon and ligaments

There is scanty of clinical evidences regarding the use of MSCs in tendon and ligament repair and regeneration. However, this clinical condition is now gaining attentions with numerous pre-clinical reports published during the last decade. Autologous undifferentiated MSCs have been shown to repair damaged tendon sans loading scaffold evident by ultrasound scanning showing optimal orientation of the repaired tendons [134]. Another study employing rat model of enthesis degeneration, 45 days post MSCs injection, showed significant healing and improved load to failure response and load required to rupture bone-tendon junction [135]. Recently, in racehorses having digital flexor tendinopathy, the safety and regenerative capacity of MSCs to reduce re-injury rate was assessed. Data suggested that after intra-lesional injections of MSCs, the re-injury rates were significantly reduced in racehorses with digital flexor tendinopathy [136]. However, from studies it not clear whether chondrocytes or MSCs contributed majorly in the production of collagen II and formation of enthesis, yet enthesis induced by MSCs was of superior nature–suggesting that MSCs were superior in regenerating enthesis than chondrocytes, nevertheless, further investigations are required to confirm that the observed clinical improvements were attributed either to the differentiation of donor derived MSCs into chondrocytes or to host derived chondrocytes induced by paracrine factors produced by donor MSCs.

To date, limited number of literature evidence is available regarding clinical use of MSCs in tendon healing and regeneration. First clinical evidence of cell reparative therapy in tendon repair did not employ MSCs rather skin derived tendon-like collagen producing skin fibroblasts were transplanted for lateral epicondylitis [137]. Likewise, number of pre-clinical and clinical studies has demonstrated the therapeutic usefulness of platelet rich plasma (PRP) in treating injured ligaments and tendons [138], yet there is no clinical evidence that demonstrated the use of MSCs in tendon and ligament repair in human subjects, despite MSCs ex-vivo differentiation into tendons.

### The demonic side of MSCs

Despite several therapeutic benefits of MSCs, plethora of literature reports support the notion of MSCs related un-wanted effects owing to their direct and indirect involvement in cancer [139]. Moreover, the role of MSCs in tumor modulation is still controversial; as many believe that MSCs can suppress tumor growth, while others ascertain that MSCs may contribute to tumor protection via anti-apoptotic effects and immune modulation. In this context, Zhu et al., demonstrated that bone marrow derived MSCs when grown in an in-vitro three dimensional tumor microenvironment enhanced the growth of ERα positive breast cancer cell lines (T47D, BT474 and ZR-75-1) without affecting the ERα negative counterpart (MDA-MB-231) [140]. Similarly, a study by Sasser et al., revealed that subcutaneous transplantation of human adipose-derived MSCs and human fetal MSCs into BALB/c-nu/nu mice alone or together with tumor cell lines F6 or SW480, supported the growth of tumor cell lines [141]. More recently, it has been demonstrated that MSCs interact with cancer stem cells (CSC) in human cancer and regulate their self-renewing capacity through cytokine networks involving IL-6 and CXCL7 [142]. Moreover evidences, such as migration of MSCs towards metastatic sites, secretion of chemokines promoting metastasis and their role in the development of drug resistance in cancer cells further confirm their demonic potential [143, 144]. Similarly, immune-modulatory role of MSCs proved to be a double edge sword benefiting patients with immune disorders while the same effect can promote cancer cells growth–evading variance checks [145]. However, a longer follow-up is required to draw a final conclusion regarding human MSCs’ tumorigenic potential, yet as per literature evidences, clinical benefits overweighs their demonic effects in clinical practice and is subject to concurrence of a tumorigenic condition.

More interestingly, studies have also shown that bone marrow derived MSCs can worsen the natural course of infection. One report by Meisel et al. showed that MSCs obtained from mice may shift the macrophages to an anti-inflammatory course, thereby suppressing inflammatory cytokine production and enhancing interleukin (IL-10) production. Animals infused by these MSCs normally resistant to *Mycobacterium tuberculosis* showed enhanced susceptibility to the disease [146].

## Conclusion

Undisputedly, MSCs offer multitude of clinical benefits in the treatment of skeletal disease along with repair and reformation of injured skeletal tissues. However, the exact mechanism entailing their in-vivo therapeutic effects needs further elucidation. Likewise, the fate of transplanted MSCs is still obscure–variably regulated by route, location and time of delivery. Moreover, from the data, reviewed above, it is reasonable to conclude that MSCs engraftment and differentiation into lineage specific cells, relevant for tissue repair, is not an absolute requirement, since numerous ensuing evidences suggest that, at some instances, even their presences as a secretory recourse is sufficient for reparative effects. More interestingly, their contribution as modulators in appeasing donor stay, while minimizing the propensity of donor rejection, is attaining much attention. However, it’s not clear that how much immune modulation is optimum for donor apt contribution in tissue repair and regeneration. Similarly, there is no agreement among researchers regarding uniform cell dose response relationship, for a particular disease or degenerative condition, suitable for lineage differentiation, release of trophic factors, immune modulation and repair supportive tissue microenvironment–linked with ex-vivo expansion of MSCs. Additionally, the presence of MSCs in many adult tissues afford technical advantage of the availability of multiple resources, however, they exhibit difference in their surface marker profiles, differentiation potential and gene expression profiles, hence not identical.

Despite tremendous progress in the field of MSCs therapeutics–affording treatments for various diseases and repair/reformation of degenerative tissues, still there are un-resolved questions that beg further investigations. Thus, the number of issues needs to be addressed before MSCs can be utilized at clinician’s precise elections–spatial and temporal control over lineage differentiation, timely release of trophic factors, homing/migration to injured or diseased site and duration of engraftment.

## Abbreviations

ADRSCs: adipose derived autologous and regenerative stem cells
ADSCs: adipose derived stem cells
ALP: alkaline phosphatase
BMP: bone morphogenetic protein
CXCL7: C-X-C motif chmokine7
ERα: estrogen receptor alpha
IGF-1: insulin-like growth factor 1
IHPP: infantile hypo-phosphatasia
IKDC: international knee documentation committee
IL10: interleukin 10
IL6: interleukin 6
IVD: inter-vertebral degeneration
MRI: magnetic resonance imaging
MSCs: mesenchymal stromal stem cells
NK: natural killer
OI: osteogenesis imperfecta
PGE-2: prostaglandin E-2
PRP: platelet rich plasma
SDF-1: stromal cell derived factor 1
TNSALP: tissue non-specific alkaline phosphatase
VEGF: vascular endothelial growth factor
WMC: whole marrow cells
